# MyD88 in hepatic stellate cells enhances liver fibrosis via promoting macrophage M1 polarization

**DOI:** 10.1038/s41419-022-04802-z

**Published:** 2022-04-28

**Authors:** Jie Zhang, Yu Liu, Haiqiang Chen, Qi Yuan, Jinyan Wang, Meng Niu, Lingling Hou, Jianchun Gu, Jinhua Zhang

**Affiliations:** 1 The College of Life Science and Bioengineering, Beijing Jiaotong University, Beijing, P.R. China; 2 Department of Immunology, Basic School of Medicine, China Medical University, Shenyang, P. R. China; 3 Department of Interventional Radiology, The First Affiliated Hospital of China Medical University, Shenyang, P. R. China; 4 Department of Oncology, Xinhua Hospital Affiliated to Shanghai Jiaotong University School of Medicine, Shanghai, P. R. China

**Keywords:** Mechanisms of disease, Kupffer cells

## Abstract

During liver fibrosis, quiescent HSCs (qHSCs) are activated to become activated HSCs (aHSCs)/myofibroblasts. The signal adapter MyD88, an essential component of TLR signaling, plays an important role in liver fibrosis. However, far less is known about the specific effects of MyD88 signaling in both qHSCs and aHSCs in the progress of liver fibrosis. Here, we used a CCl_4_-induced mouse fibrosis model in which MyD88 was selectively depleted in qHSCs (GFAP^MyD88−/−^ mice) or aHSCs (α-SMA^MyD88−/−^ mice). MyD88 deficiency in qHSCs or aHSCs attenuated liver fibrosis in mice and inhibited α-SMA-positive cell activation. Inhibition of MyD88 in HSCs decreased α-SMA and collagen I levels, inflammatory cell infiltration, and pro-inflammatory gene expression. Furthermore, MyD88 signaling in HSCs increased the secretion of CXCL10, which promoted macrophage M1 polarization through CXCR3, leading to activation of the JAK/STAT1 pathway. Inhibition of CXCL10 attenuated macrophage M1 polarization and reduced liver fibrosis. Thus, MyD88 signaling in HSCs crucially contributes to liver fibrosis and provides a promising therapeutic target for the prevention and treatment of liver fibrosis.

## Introduction

Hepatic fibrosis is a dynamic process characterized by the accumulation of excessive amounts of extracellular matrix (ECM) resulting from chronic liver injury of several types of etiology, including chronic viral infection, alcoholic liver disease (ALD), non-alcoholic steatohepatitis, and chemical injury [1, 2]. Although fibrosis can reverse after eliminating the cause of injury, chronic injury left untreated can lead to cirrhosis. Acute decompensation (AD) of cirrhosis is a major cause of mortality and is associated with increased individual risk of hepatocellular carcinoma (HCC) worldwide [3, 4]. Although some drugs have antifibrotic effects in animal models, they are rarely used in clinical practice. Therefore, it is highly desirable to further explore the pathogenesis of liver fibrosis and discover new therapeutic targets for liver fibrosis.

The hepatic stellate cells (HSCs) which represent up to 10% of all resident liver cells, express glial fibrillary acidic protein (GFAP) and/or desmin [1]. In normal physiological conditions, HSCs contain retinoid lipid droplets and exhibit a quiescent phenotype. During liver injury, quiescent HSCs (qHSCs) downregulate the expression of vitamin A, GFAP and become activated HSCs (aHSCs), which are characterized by expression of α-smooth muscle actin and differentiation into proliferating myofibroblast-like cells [5, 6]. Thus, activated HSCs become the major cell type responsible for hepatic fibrosis [7].

Immune cells, including macrophages, play a key role in the process of liver injury and subsequent liver fibrosis [1, 8, 9]. Activated macrophages are usually divided into two groups, the classical activated inflammatory phenotype (M1) and the non-classical activated anti-inflammatory phenotype (M2) [10, 11]. M1 macrophages secrete various pro-inflammatory cytokines, such as IL-6 and TNF-α, which promote liver inflammation and liver injury. M2 macrophages synthesize anti-inflammatory cytokines, such as IL-10 and TGF-β, which remodel damaged tissues and accelerate the repair process [12]. Imbalance between M1 and M2 macrophages mediates the progression and regression of liver fibrosis. In the early stages of liver injury, bone marrow-derived mononuclear cells accumulate extensively in the liver and then differentiate into inflammatory macrophages (mainly M1 macrophages) to produce pro-inflammatory and pro-fibrotic cytokines leading to promotion of hepatic inflammatory responses and activation of HSCs [13, 14].

Toll-like receptors (TLRs) are the “gatekeepers” of the immune system in humans and other animals to protect the host from invading bacteria, viruses, and other microorganisms [15]. Myeloid differentiation primary response gene 88 (MyD88) is a common adaptor molecule that transmits signals from members of both the TLR (except TLR3) and IL-1 receptor families [16]. There are increasing evidence that TLRs and MyD88 play important roles in liver fibrosis. TLR9 signaling promotes steatohepatitis and fibrosis through induction of IL-1β [17]. TLR4 enhances TGF-β signaling and promotes hepatic inflammation and fibrosis, which also contributed to the development of HCC [18, 19]. Since MyD88 is widely expressed in a variety of cells in the liver, including hepatocytes, HSCs and Kupffer cells, etc, increasing evidence have suggested that MyD88 plays different roles under different circumstances in different cell types. Recently, it has been reported that deletion of MyD88 in B cells attenuated CCl_4_-induced liver fibrosis [20]. We also found MyD88 in macrophages enhance liver fibrosis by activation of NLRP3 inflammasome in HSCs [21].In addition, MyD88 expression was crucial for HSC activation in vitro [22]. However, far less is known about the function of MyD88 signaling-specific deficiency in HSCs in a fibrosis model. In the present study, we showed that HSC specific MyD88 deletion inhibited the activation of HSCs and attenuated CCl_4_-induced liver fibrosis. Furthermore, CXCL10 secreted from HSCs promotes macrophage M1 polarization via activating JAK/STAT1 pathway, which in turn promotes liver fibrosis. Thus, MyD88 in HSCs may represent a potential target for anti-fibrotic strategies in therapies.

## Results

### MyD88 expression in myofibroblasts is upregulated during the progression of liver fibrosis

**Fig. 1:**
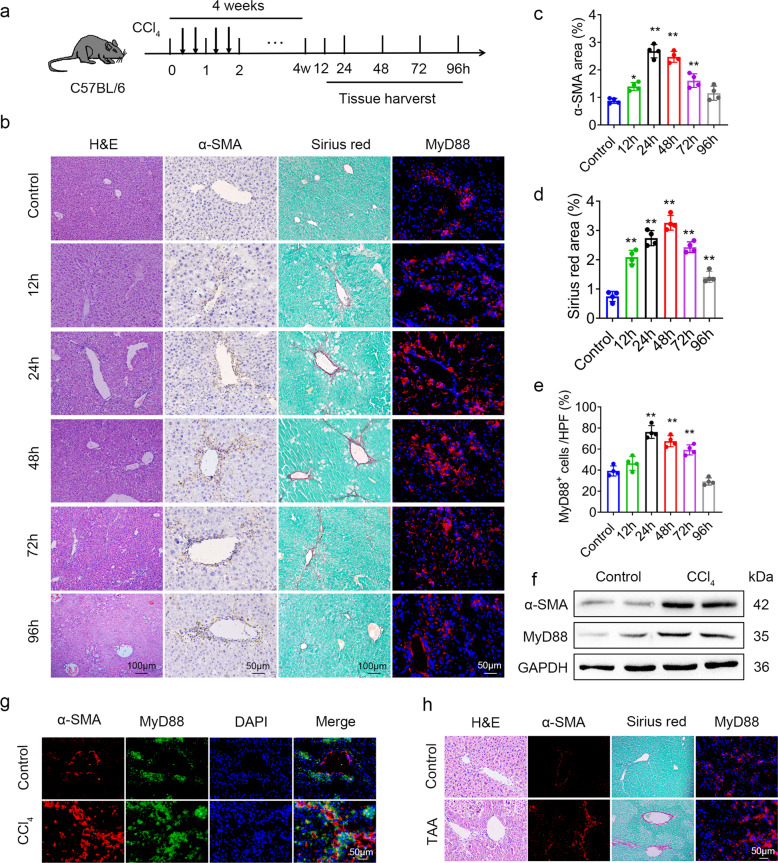
MyD88 in HSCs is activated during the progression of liver fibrosis. Groups of C57BL/6 mice (*n* = 4 per group) were subjected to CCl_4_-induced liver fibrosis. Data were representatives of at least three independent experiments. These charts show the average of the experimental results, with the error bar showing the standard error of mean. **a** Schematic illustration of CCl_4_-induced liver fibrosis. **b** Liver tissues were harvested at the indicated timepoints and stained for H&E, Sirius Red, anti-α-SMA and MyD88 antibodies. Scale bar, 50 or 100 μm. **c**–**e** Statistical analysis of the α-SMA, Sirius red, and MyD88 staining in liver tissues. *p < 0.05 and **p < 0.01. **f** The expression levels of α-SMA and MyD88 proteins in liver tissues were determined by Western blot analysis. **g** Representative double-staining of α-SMA and MyD88 in liver tissues. Scale bar, 50 μm. **h** Sections of liver tissues were stained for H&E, Sirius red, α-SMA and MyD88 in C57BL/6 mice treated with TAA. Scale bar, 50 μm.

To investigate the role of MyD88 during liver fibrosis, C57BL/6 mice were administered with carbon tetrachloride (CCl_4_), a reversible model, widely used for the study of both progression and resolution of liver fibrosis [23]. Liver tissues were harvested at different time points after liver fibrosis was established as shown in Fig. 1a. HSC activation and MyD88 expression were examined by immunohistochemical staining with α-SMA (a marker for activated HSCs) and collagen Ι. As shown in Fig. 1b, the HSCs were activated by CCl_4_. The number of SMA^+^ cells is increased significantly in the liver continued for the first 72 h (progression), followed by a gradual degradation of collagen at 96 h (resolution) (Fig. 1c). The collagen deposition was also significantly increased in livers during the progression phase as indicated by Sirius Red staining (Fig. 1b, d). In accordance with HSC activation in the liver, the expression of MyD88 peaked at 24 h after the final CCl_4_ injection, remained significantly high during the progression phase, and dropped rapidly to the control level during the resolution phase at 96 h. MyD88 expression in liver fibrosis was further confirmed by Western blot analysis (Fig. 1f). Double immunofluorescence staining revealed that MyD88 was highly expressed in α-SMA^+^ myofibroblasts in the fibrotic liver tissues (Fig. 1g). In addition, upregulation of MyD88 during fibrosis progression was also found in thioacetamide (TAA) induced liver fibrosis model (Fig. 1h), which based on C57BL/6 mice treated with TAA [24]. The obtained results suggest that the upregulation of MyD88 in liver is a common phenomenon during tissue repair, a process that is independent of the mouse model used.

### Hepatic stellate cell-specific MyD88 deletion attenuates CCl_4_-induced liver fibrosis

**Fig. 2:**
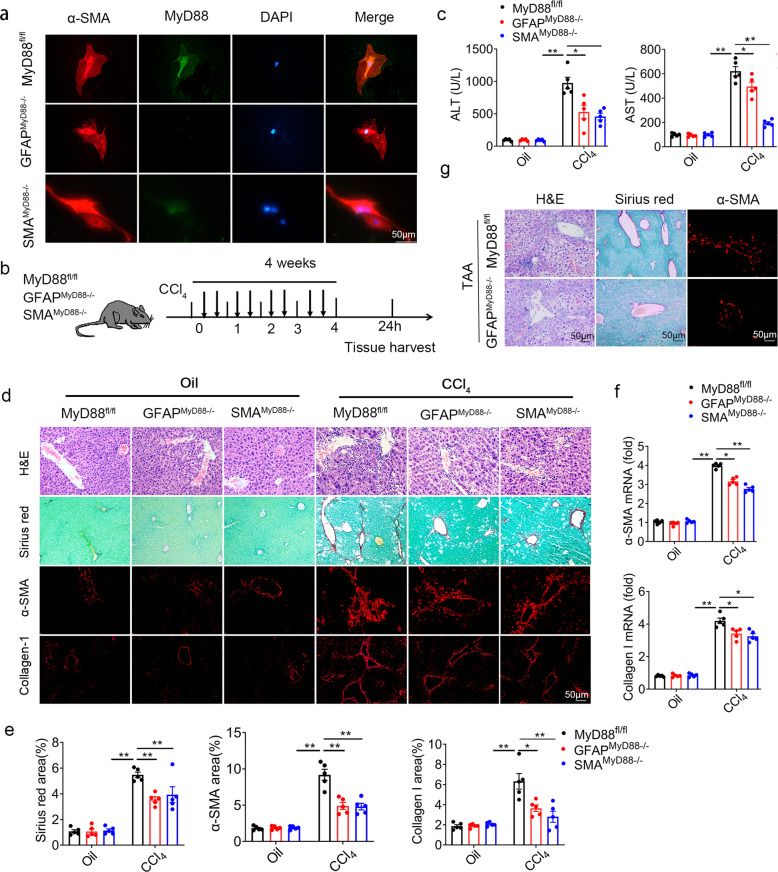
MyD88 deficiency in HSCs attenuates CCl_4_-induced liver fibrosis. **a** HSCs were isolated from the liver of mice in the MyD88^fl/fl^ group and the GFAP^MyD88−/−^, SMA^MyD88−/−^ groups, respectively, and were induced into myofibroblasts after 7 days of culture for α-SMA (red) and MyD88 (green) double immunofluorescence staining. Scale bar, 50 μm. Data were representatives of at least three independent experiments. **b** Groups of MyD88^fl/fl^, GFAP^MyD88−/−^, SMA^MyD88−/−^ mice (*n* = 5 per group) were subjected to the CCl_4_-induced liver fibrosis. Data were representatives of at least three independent experiments. **c** Serum ALT and AST levels. *p < 0.05 and **p < 0.01. **d** Representative staining of H&E, Sirius red, collagen I and α-SMA (Scale bar, 50 μm) in liver tissues. **e** Statistical analysis of the Sirius red, collagen I and α-SMA staining in liver tissues. *p < 0.05 and **p < 0.01. **f** mRNA levels of α-SMA and collagen I in liver tissues. *p < 0.05 and **p < 0.01. **g** Groups of MyD88^fl/fl^ and GFAP^MyD88−/−^ mice (*n* = 5 per group) were subjected to the TAA-induced liver fibrosis. Representative staining of H&E, Sirius red, and α-SMA (Scale bar, 50 μm) in liver tissues. These charts show the average of the experimental results, with the error bar showing the standard error of mean.

To further explore the role of MyD88 in HSCs, we crossed mice carrying the loxP-flanked MyD88 allele [25] with GFAP-cre mice [26] or α-SMA-cre mice [27] to achieve MyD88 ablation specifically in qHSCs or aHSCs. The resulting GFAP-cre-MyD88^flox/flox^ (GFAP^MyD88−/−^) and α-SMA-cre-MyD88^flox/flox^ (SMA^MyD88−/−^−) mice were born at the expected Mendelian ratio, and were viable and fertile. Their single-transgenic littermates were used as control. The absence of MyD88 in HSCs from the GFAP^MyD88−/−^ or SMA^MyD88−/−^ mice was demonstrated by double immunofluorescence staining (Fig. 2a). To identify the role of MyD88 in HSCs in liver fibrosis, we treated GFAP^MyD88−/−^−, SMA^MyD88−/−^− mice and control littermates with CCl_4_ (Fig. 2b). The serum levels of ALT and AST were significantly decreased in CCl_4_-treated GFAP^MyD88−/−^and SMA^MyD88−/−^ mice, compared with CCl_4_-treated control mice (Fig. 2c). In addition, CCl_4_-treated GFAP^MyD88−/−^ and SMA^MyD88−/−^ mice were found to exhibit attenuated liver injury and liver fibrosis by hematoxylin and eosin (H&E) staining and Sirius red staining (Fig. 2d). Next, we performed immunofluorescence staining in liver tissues, consistently, both the area of type I collagen^+^ and α-SMA^+^ cells are reduced in the liver of CCl_4_-treated GFAP^MyD88−/−^ and SMA^MyD88−/−^ mice (Fig. 2d, e). There were no differences in HE, Sirius red staining as well as type I collagen expression in liver tissues of GFAP^MyD88−/−^−, SMA^MyD88−/−^ mice and control mice treated with corn oil (Fig. 2d, e). The mRNA levels of α-SMA and type I collagen were downregulated in liver tissues of CCl_4_-treated GFAP^MyD88−/−^/SMA^MyD88−/−^ mice compared with the control group (Fig. 2f). These results indicated that specific knockdown of MyD88 in both quiescent HSCs and activated HSCs attenuated CCl_4_-induced liver fibrosis. In addition, similar results were also found in TAA induced liver fibrosis model (Fig. 2g).

### HSC-specific MyD88 deletion attenuates liver inflammatory response

**Fig. 3:**
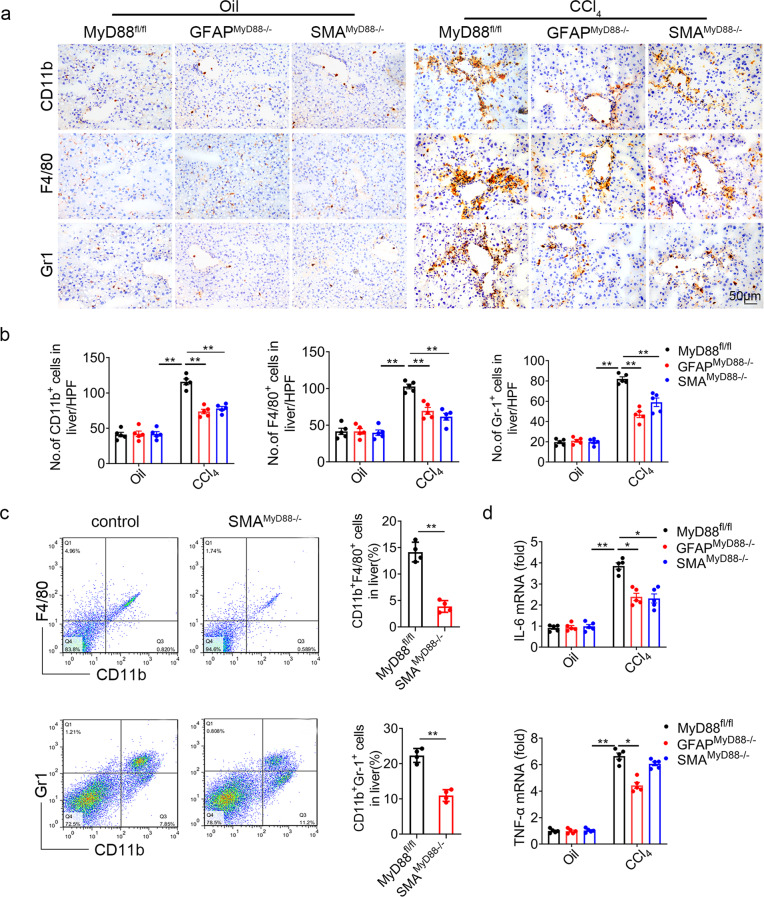
Deficiency of MyD88 in HSCs attenuates the CCl_4_-induced liver inflammatory response. Groups of MyD88^fl/fl^, GFAP^MyD88−/−^, SMA^MyD88−/−^ mice (*n*= 5 per group) were subjected to the CCl_4_-induced liver fibrosis. Data were representatives of at least three independent experiments. These charts show the average of the experimental results, with the error bar showing the standard error of mean. **a** Representative staining of CD11b, F4/80 and Gr-1 in liver tissues (Scale bar, 50 μm) and (**b**) statistical analysis (magification, 200×). *p < 0.05, **p < 0.01. **c** Isolation of liver lymphocytes from CCl_4_-induced MyD88^fl/fl^ and SMA^MyD88−/−^ mice and Flow cytometry analysis of the proportion of CD11b^+^F4/80^+^ macrophages and CD11b^+^Gr-1^+^ neutrophils in the livers after staining with F4/80, CD11b, and Gr-1 antibodies, **p < 0.01. **d** The mRNA levels of IL-6 and TNF-α in liver tissues were measured using qPCR analysis, *p < 0.05, **p < 0.01.

We next explored whether MyD88 signaling in HSCs during fibrosis regulates the inflammatory response in the liver. We performed immunohistochemical staining of liver tissues and found although there were no significant differences in the liver tissue of corn oil treated mice, the infiltration of CD11b^+^ macrophages, F4/80^+^ macrophages and Gr1^+^ neutrophils were prominently increased in both GFAP^MyD88−/−^and SMA^MyD88−/−^ mice after CCl_4_ treatment in comparison to that of their littermate controls (Fig. 3a, b). FACS analysis also showed similar results (Fig. 3c). The percentages of both CD11b^+^F4/80^+^cells and CD11b^+^Gr1^+^ cells in intrahepatic lymphocytes in the livers of SMA^MyD88−/−^ mice were decreased significantly compared with that in WT mice (17.7 ± 0.97% vs. 11 ± 0.84%) (Fig. 3c). In addition, the mRNA expression levels of pro-inflammatory cytokines IL-6, TNF-α were also significantly reduced in the livers of CCl_4_-induced GFAP^MyD88−/−^ and SMA^MyD88−/−^ mice (Fig. 3d). These results indicated that specific knockdown of MyD88 in HSCs attenuated the CCl_4_-induced inflammatory response in the liver.

### Inhibition of MyD88 attenuates HSC activation and inflammatory response

**Fig. 4:**
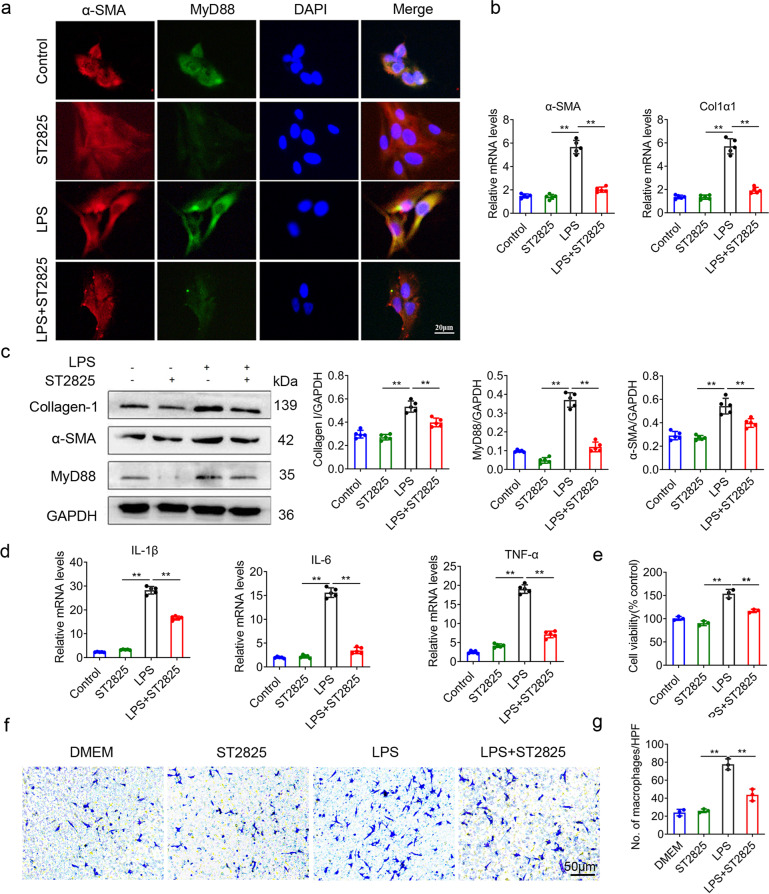
Inhibition of MyD88 attenuates the activation and inflammatory response of hepatic stellate cells. LX-2 cells were activated with LPS (100 ng/mL) and incubated with MyD88 inhibitor ST2825 (20 μM) for 24 h. **a** Immunofluorescence staining for α-SMA (red) and MyD88 (green) in LX-2 cells. Scale bar, 20 μm. **b** The mRNA levels of activation-related genes α-SMA, Col1α1 in LX-2 cells were measured using qPCR analysis, *p < 0.05, **p < 0.01. **c** The protein levels of MyD88, α-SMA and collagen I in LX-2 cells were detected by Western blot. The densities of proteins were quantified by densitometry. α-SMA and MyD88 were normalized to GAPDH. *p < 0.05, **p < 0.01. **d** The mRNA levels of IL-1β, IL-6, and TNF-α in LX-2 cells were detected by qPCR, **p < 0.01. **e** CCK8 assay for cell viability. **p < 0.01. **f** Chemotactic activity was analyzed to evaluate the role of MyD88 in LX-2 on the migration of macrophages as described in Materials and Methods. Macrophages migrated toward LX-2, ST2825-treated LX-2, LPS-treated LX-2, LPS, and ST2825-treated LX-2 are shown. And the mean numbers of cells migrated through the filter per HPF (×400) are shown (**g**). Data were representatives of at least three independent experiments. Scale bar, 50 μm. **p < 0.01. These charts show the average of the experimental results, with the error bar showing the standard error of mean.

To further investigate the role of MyD88 in HSC activation in vitro, the HSC LX-2 was activated with LPS (100 ng/mL) and MyD88 signaling was inhibited with the inhibitor ST2825 (20 μM). LPS-treated HSCs showed a typical myofibroblast-like morphology with a more widely spread pattern at day 4 in culture (Fig. 4a). Staining for α-SMA showed that there were more α-SMA positive cells at day 4 upon LPS stimulation (Fig. 4a), which also expressed MyD88. After MyD88 signaling inhibited with ST2825, HSC activation was also decreased shown by immunofluorescence staining (Fig. 4a). In addition, the mRNA levels of a-SMA and collagen I significantly decreased in HSCs after MyD88 signaling was inhibited by ST2825 (Fig. 4b). The decreased protein levels of a-SMA, collagen I and MyD88 were further demonstrated by Western blot analysis (Fig. 4c). The HSC LX-2 was also activated with TGFβ, and the mRNA levels of a-SMA and collagen I significantly decreased after MyD88 signaling was inhibited (Fig. S1a). These results indicated that inhibition of MyD88 attenuated HSC activation.

Although LPS activation of HSCs promoted the expression of inflammatory factors, the mRNA expression levels of IL-1β, IL-6 and TNF-α were significantly reduced after inhibition of MyD88 signaling by ST2825 (Fig. 4d). The similar result was obtained with LX-2 treated with TGFβ and ST2825 (Fig. S1b). Detected by CCK, the HSC cell proliferation was decreased remarkably after ST2825 treatment (Fig. 4e). Then Transwell assays were performed to further elucidate the role of MyD88 signaling in HSCs in macrophage recruitment. Only a small number of macrophages were found to migrate toward DMEM. LPS stimulation of LX-2 significantly increased the migration of macrophages. Furthermore, the increased migration of macrophages could be blocked by MyD88 inhibitors (Fig. 4f). The mean numbers of macrophages migrated through the filter were shown in Fig. 4g. Together, these results indicated that MyD88 signaling promotes HSC activation, inflammatory response, and macrophage recruitment contributing to liver inflammation and fibrosis.

### Specific genetic deletion of MyD88 in HSCs reduces CXCL10 secretion

**Fig. 5:**
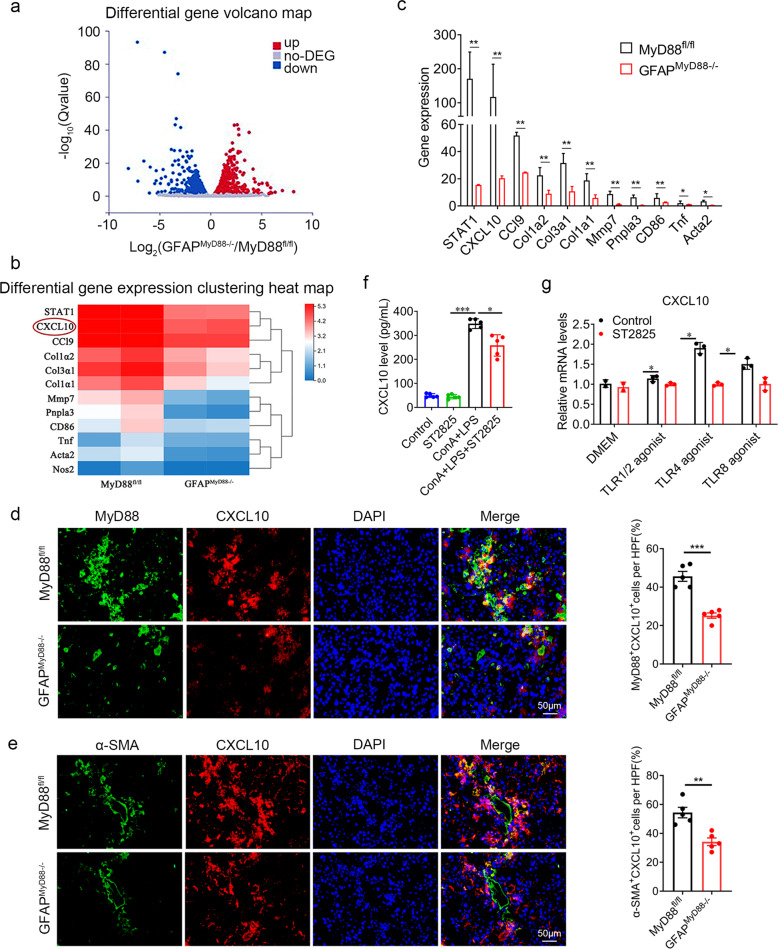
Secreted CXCL10 from HSCs was critical for liver fibrosis. RNA sequencing analysis of DEGs between CCL_4_-induced liver tissues from MyD88^fl/fl^ and GFAP^MyD88−/−^ mice. **a** Volcano diagram of DEGs, threshold is padj < 0.05 and the absolute value of log2 Ratio≥1. **b** Heatmap view of gene expression of fibrosis and inflammation related markers. **c** Fibrosis-related gene expression in liver tissues. **d** Double staining for MyD88 and CXCL10 in liver tissues (Scale bar, 50 μm) and statistical analysis. ***p < 0.001. **e** Double staining for α-SMA and CXCL10 in liver tissues (Scale bar, 50 μm). **p < 0.01. **f** The secretory protein levels of CXCL10 in LPS, ConA and inhibitor ST2825 treated LX-2 medium were measured using ELISA. *p < 0.05, ***p < 0.001; **g** Inhibited MyD88 in LX-2 cells were treated with Inhibitor ST2825 (Scale bar, 20 μM), and agonists of TLR1/2, TLR4, TLR8 for 24 h. CXCL10 expression in LX-2 cells was detected by qPCR. **p < 0.01. These charts show the average of the experimental results, with the error bar showing the standard error of mean.

To further compare detailed changes of the gene expression signature, we performed protein-coding mRNA-seq analysis of liver tissues from CCl_4_ treated GFAP^MyD88−/−^ mice and control mice. A total of 1149 differentially expressed genes (DEGs) were identified, including 607 up-regulated and 542 down regulated genes (Fig. 5a; padj <0.05 and the absolute value of log2 Ratio≥1). Interestingly, GFAP^MyD88−/−^ mice had lower expression of genes related with liver fibrosis and inflammation, compared with control mice (Fig. 5b). Especially, the expression of CXCL10 was decreased most significantly in CCl_4_-treated GFAP^MyD88−/−^ mice compared with control mice (Fig. 5b, c). Decreased number of CXCL10^+^ cells were also demonstrated by immunofluorescence double staining in MyD88 positive area of liver tissues (Fig. 5d). Further double immunofluorescence staining revealed that CXCL10 was highly expressed in α-SMA^+^ cells in the fibrotic liver tissues (Fig. 5e), demonstrating that CXCL10 is expressed in aHSCs. Consistently, secretion level of CXCL10 protein was significantly increased in LX-2 cells induced by LPS/ConA, while the MyD88 inhibitor ST2825 greatly suppressed COA and LPS-induced CXCL10 protein expression level in HSCs (Fig. 5f). Toll-like receptors in HSCs were activated in vitro by different Toll-like receptor activators. Next, we tested whether stimulation of MyD88-dependent TLRs modulates CXCL10 expression in HSCs. Stimulation of LX-2 with the bacterial agonists for TLR4 (LPS), TLR1/2 (Pam3CSK4) and TLR8 (motolimod) resulted in downregulation of CXCL10, which is due to inhibition of MyD88 with inhibitor ST2825 (Fig. 5g). TLR8 agonist and LPS have similar effects to promote the expression of CXCL10 in LX-2 depending on the MyD88 pathway. In contrast, the agonist for TLR7 (imiquimod) did not affect CXCL10 expression due to deletion of MyD88 (Fig. 5g). Among these TLR agonists, LPS had the most significant effect on expression of CXCL10 in LX-2, and we further studied LPS-induced LX-2. These results suggest that MyD88-dependent TLR signaling promotes CXCL10 expression in HSCs.

### CXCL10 promotes M1 polarization in macrophages via activating JAK/STAT1 pathway

**Fig. 6:**
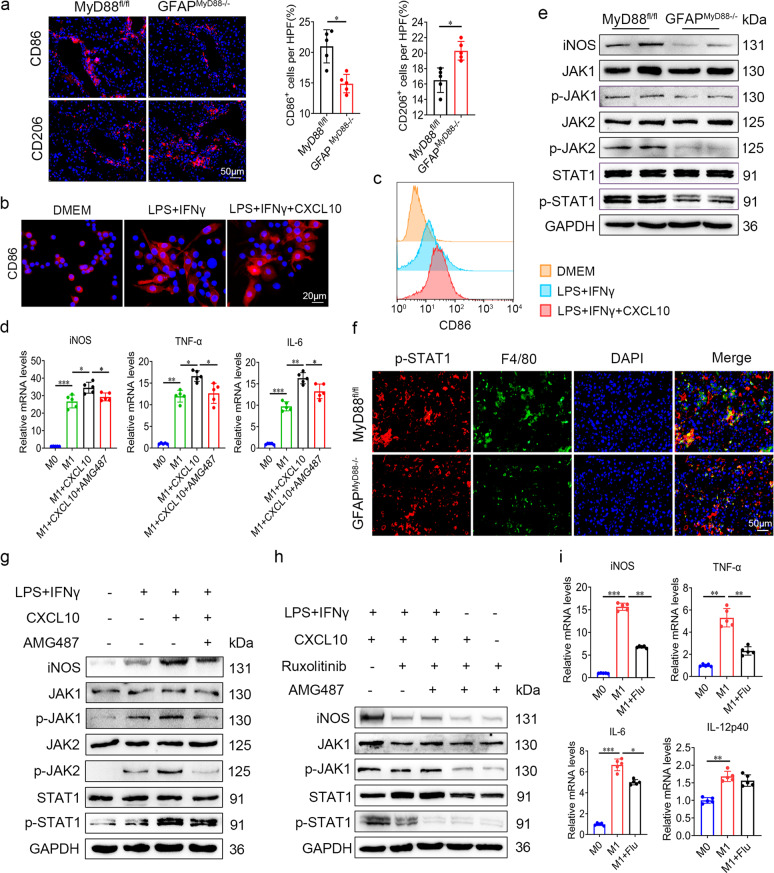
CXCL10 promotes M1 polarization in macrophages via activating JAK/STAT1 pathway. **a** Immunofluorescence staining for CD86 and CD206 in liver tissues of CCl_4_ induced MyD88^fl/fl^, GFAP^MyD88−/−^ mice and statistical analysis. Scale bar, 50 μm. *p < 0.05. RAW264.7 cells was induced by LPS and IFNγ, 10 ng/mL CXCL10 recombinant protein. **b** Immunofluorescence staining for CD86 in Raw264.7 cells. **c** Flow cytometry analysis of the proportion of CD86^+^ cells treated with LPS, IFNγ and CXCL10 recombinant protein. **d** RAW264.7 cells was induced by LPS and IFNγ, 10 ng/mL CXCL10 recombinant protein, and 500 nM inhibitor of CXCL10 receptor CXCR3 AMG 487 for 24 h. mRNA expression of iNOS, IL-6 and TNF-α in RAW264.7 cells were detected by qPCR. *p < 0.05, **p < 0.01, ***p < 0.001. **e** Protein levels of p-JAK, JAK, p-STAT1, STAT1 and iNOS in CCl_4_ treated liver tissues of MyD88^fl/fl^ and GFAP^MyD88−/−^ mice were examined by Western blot. **f** Double immunofluorescence staining for F4/80, p-STAT1 in liver tissues of CCl_4_ treated MyD88^fl/fl^ and GFAP^MyD88−/−^ mice. Scale bar, 50 μm. **g** Protein levels of iNOS, p-JAK1, JAK1, p-JAK2, JAK2, p-STAT1, and STAT1 in LPS, IFNγ, CXCL10 and AMG487 treated RAW264.7 cells were detected by Western blot. **h** Protein levels of iNOS, p-JAK, JAK, p-JAK2, JAK2, p-STAT1 and STAT1 in LPS, IFNγ, CXCL10, ruxolitinib and AMG487 treated RAW264.7 cells were detected by Western blot. **i** RAW264.7 cells were treated with LPS, IFNγ and 10 μM Fludarabine for 24 h to inhibit STAT1 phosphorylation. mRNA expression of M1-type macrophage-related genes, iNOS, IL-6, TNF-α and IL-12p40 was detected by qPCR, *p < 0.05, **p < 0.01, ***p < 0.001. Data were representatives of at least three independent ex*p*eriments. These charts show the average of the experimental results, with the error bar showing the standard error of mean.

In Fig. 4f, g, we have demonstrated that MyD88 signaling in HSCs LX-2 promotes inflammatory responses and macrophage recruitment. From the mRNA-seq analysis of liver tissues in Fig. 5b, c, we also found that the expression of CD86 and iNOS in GFAP^MyD88−/−^ mice were decreased significantly compared with that in control mice, which suggests that macrophage M1 polarization may be inhibited. Then we analyzed the number of CD86^+^ and CD206^+^ macrophages in liver tissues and found that GFAP^MyD88−/−^ mice exhibited a reduction in the M1-like phenotype and an increase in the M2-like phenotype compared to that in control mice with liver fibrosis (Fig. 6a). Based on these observations in vivo, we next analyzed the HSC-specific role of MyD88 on macrophage polarization in vitro. Since deletion of MyD88 in HSCs decreased the secretion of CXCL10, we wonder if MyD88 in HSCs promotes the polarization of macrophages through CXCL10. Then macrophage RAW264.7 cells were cultured with LPS and IFNγ for 24 h to induce M1 polarization. CD86 expression was analyzed by immunostaining. LPS and IFNγ activated the expression of CD86 in macrophages compared with untreated DMEM group, and CXCL10 further upregulated the expression of CD86 in macrophages (Fig. 6b, c). In addition, we found the levels of iNOS, IL-6 and TNF-α were upregulated significantly compared to the untreated group. Exogenous addition of CXCL10 recombinant protein further promoted M1 polarization of macrophages. However, in RAW264.7 cells treated with AMG487, an inhibitor of CXCL10 receptor CXCR3, abolished CXCL10 induced iNOS, IL-6 and TNF-α levels (Fig. 6d). The results indicated that deletion of MyD88 in HSCs reprogrammed the macrophages and downregulated M1-related genes.

Expression of the members of the signal transducer and activator of transcription (STAT) family have been linked with macrophage polarization. Higher levels of STAT1 are found in M1 macrophages [28]. From the RNA Seq results (Fig. 5b), we found that expression of STAT1 was decreased significantly after MyD88 deletion, suggesting that CXCL10 may induce macrophage M1 polarization via promoting STAT1 phosphorylation. To elucidate the mechanism whereby HSC-derived MyD88 affects macrophage polarization, we analyzed CCl_4_ induced liver tissues by Western blot. GFAP^MyD88−/−^ mice had suppressed IFNγ-induced JAK1/2 and STAT1 phosphorylation related to the inhibition of iNOS protein compared with that in control mice (Fig. 6e). Moreover, with further analysis of immunofluorescence, we found that the expression of p-STAT1 in macrophages was reduced significantly in liver tissues in GFAP^MyD88−/−^ mice (Fig. 6f). Furthermore, after treatment with exogenous addition of CXCL10 recombinant protein, the protein levels of p-JAK1/2 and p-STAT1 in RAW264.7 cells were greatly increased. However, the simultaneous addition of CXCL10 recombinant protein and AMG 487 (an inhibitor of CXCL10 receptor CXCR3) abolished the expression of p-STAT1 (Fig. 6g). Moreover, the inhibition of JAK activities by the JAK inhibitor ruxolitinib completely abolished iNOS expression in RAW264.7 cells (Fig. 6h), suggesting that JAK-STAT1 signaling is responsible for M1 polarization. Furthermore, macrophages were treated with the STAT1 inhibitor Fludarabine. As shown in Fig. 6i, the addition of Fludarabine to LPS-induced RAW264.7 cells further downregulated M1-related genes, such as IL-6, iNOS and TNF-α. Taken together, the above data demonstrated that HSCs derived CXCL10 promoted macrophage M1 polarization possibly by activating JAK-STAT1 signaling.

### Targeting CXCL10 by CXCL10-neutralizing antibody attenuates CCl_4_ induced liver fibrosis

**Fig. 7:**
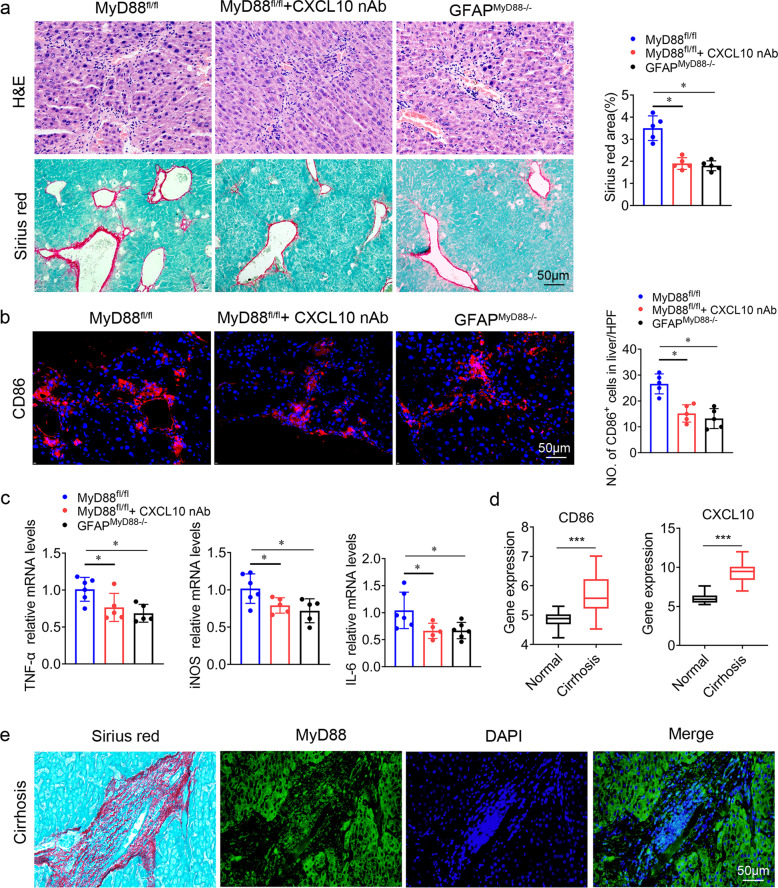
Anti-CXCL10 treatment attenuates CCl_4_-induced liver fibrosis. Groups of MyD88^fl/fl^ mice (*n* = 5 per group) were treated with CCl_4_ and anti-CXCL10 mAb or mIgG twice a week for 2 weeks. GFAP^MyD88−/−^mice were injected with CCl_4_ at the same time. These charts show the average of the experimental results, with the error bar showing the standard error of mean. **a** H&E and Sirius red staining of liver tissues. Scale bar, 50 μm. *p < 0.05. **b** immunofluorescence staining of CD86 in liver tissues. Scale bar, 50 μm, *p < 0.05. **c** The mRNA levels of IL-6 and iNOS in LX-2 cells were detected by qPCR, *p < 0.05. **d** Analysis of CXCL10 and CD86 expression in clinical samples from normal and cirrhosis patients in the GSE14323 database. Differences were analyzed by the log-rank test. ***p < 0.001. **e** Consecutive sections of liver tissues of cirrhosis patients for Sirius red and MyD88 immunofluorescence staining.

Since CXCL10 plays an important role in liver fibrosis pathogenesis, we examined the therapeutic value of CXCL10 neutralizing antibody in mouse models. By administering CXCL10 neutralizing antibody (Invitrogen, CA, USA) or an isotypic control (mIgG) preventively to the CCl_4_-treated mice, we found that CXCL10 neutralizing antibody significantly attenuates CCl_4_ induced liver fibrosis as indicated by reduced collagen deposition in whole liver lysate (Fig. 7a). The similar result was also showed by Western blot (Fig. S2). More importantly, the number of CD86^+^ cells were markedly reduced after neutralization of CXCL10 (Fig. 7b). mRNA levels of iNOS and IL-6 were significantly decreased after CXCL10 neutralization (Fig. 7c). Similarly, the protein level of iNOS was significantly downregulated after CXCL10 neutralization (Fig. S2). These results indicated that targeting CXCL10 by CXCL10-neutralizing antibodies inhibited the progression of liver fibrosis via attenuating macrophage M1 polarization. Through the analysis of the expression of CXCL10 and CD86 in normal and cirrhotic clinical samples in GEO database, we found that CXCL10 expression was increased in cirrhotic tissues compared to normal liver along with increased expression of the M1-type related gene CD86 in macrophages (Fig. 7d). Furthermore, two adjacent paraffin sections of cirrhotic tissues were stained with Sirius red and MyD88 immunofluorescence. Interestingly, we observed many MyD88^+^ cells, which accumulated around fibrotic areas in human cirrhotic liver tissues (Fig. 7e). Together, these data suggest that CXCL10 may be a promising therapeutic target for the prevention and treatment of liver fibrosis.

## Discussion

**Fig. 8:**
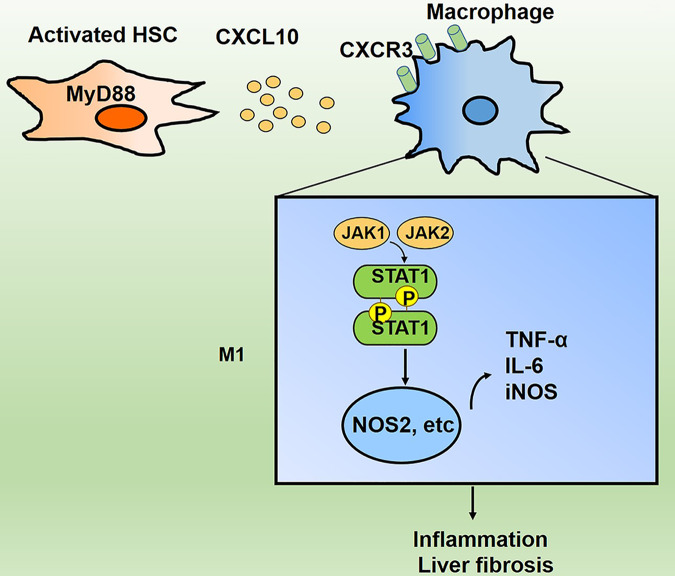
Schematic of MyD88 signaling in HSCs in fibrosis model. MyD88 in HSCs promotes CXCL10 secretion. CXCL10 further induces macrophage M1 polarization through activation of JAK/STAT1 signaling pathway, which in turn promotes liver inflammatory response and fibrosis.

Liver fibrosis and its end-stage cirrhosis, represent major health problems worldwide. MyD88 is a key player in the process of liver fibrosis. However, due to its wide expression, the cell-type specific contribution of MyD88-mediated signaling to liver fibrosis is still unclear. In this study, we found that MyD88 deficiency in HSCs attenuates liver fibrosis and inflammatory cell infiltration in CCl_4_-induced mice. Furthermore, mechanistic studies showed that MyD88 deficiency in HSCs reduced CXCL10 expression. Exogenous addition of CXCL10 recombinant protein could induce macrophage M1 polarization through activation of STAT1 signaling pathway, which in turn promoted liver inflammatory response and fibrosis (Fig. 8). Thus, MyD88 in HSCs may represent a potential candidate for the prevention and therapy of liver fibrosis.

Hepatic fibrosis is a pathological process characterized by a reversible accumulation of collagenous matrix and sustained inflammation [29]. Increasing evidence supports that activation of MyD88 is related to the pathogenesis of fibrosis-related diseases [30, 31]. HSCs/myofibroblasts are a major source of fibrogenic cytokines and extracellular matrix [29]. In this study, we first demonstrated the role of MyD88 signaling in HSCs in liver fibrosis. Our results showed that the MyD88 signaling pathway was activated in HSCs and promoted CCl_4_-induced liver fibrosis, which is consistent with previous studies that LPS/TLR4/MyD88 signaling is involved in the activation of HSCs during liver injury [32, 33].

Hepatic stellate cells (HSCs) activation results from the inflammatory activity of liver immune cells, predominantly macrophages. Macrophage-derived TGF-β activates hepatic stellate cells and is the most potent known fibrogenic agonist [6]. Hepatic macrophages, including Kupffer cells and recruited macrophages, also enhance liver fibrosis by promoting the survival of HSCs in a NF-κB–dependent manner [9]. Macrophage/HSCs crosstalk is mediated by IL-1 and TNF-α. Neutralization of these cytokines in co-culture experiments or genetic ablation of their receptors in mouse models led to decreased fibrosis and increased apoptosis of HSCs [34]. Consistently, inhibition of NF-κB with sulfasalazine stimulated apoptosis of activated rat and human HSCs, impairing hepatic fibrosis [7].

During liver fibrosis, in addition to activation of Kupffer cells, many monocytes are recruited to the damaged liver, and they play an important role in the process of liver fibrosis [1]. We found that MyD88 signaling in HSCs promoted the recruitment of macrophages (Fig. 3a, b). During liver injury, macrophages are divided into “pro-inflammatory” M1 and “anti-inflammatory” M2 types to adapt to the local microenvironment [35]. Increased intestinal permeability leads to elevated LPS levels during liver disease, which promote HSC activation [36, 37]. Activated HSCs secrete a variety of cytokines, including pro-inflammatory factors and fibrosis-related factors [7]. IL-4 upregulates histone demethylase-dependent chromatin modifications in hepatic macrophages, thereby inhibiting M1-type macrophages, promoting M2-type macrophages, and significantly reducing liver inflammation [38]. In the present study, we found that MyD88 signaling in HSCs promoted inflammatory response and macrophage recruitment, while inhibition of MyD88 in HSCs resulted in decreased expression of CD86 and increased expression of CD206 in macrophages (Fig. 6a). These results demonstrate that MyD88 in HSCs plays an important role in promoting macrophage M1 polarization, which in turn promotes the inflammatory response and fibrosis in the liver. Therefore, targeting macrophage polarization is a novel therapeutic approach. During liver injury, HSCs produce chemokines. In CCl_4_ or FFC-induced liver injury and liver fibrosis, CXCL10 can promote liver fibrosis and infiltration of inflammatory macrophages [39]. In this study, we found that liver injury and collagen deposition were reduced after CXCL10 neutralization in mice, which attenuated CCl_4_-induced liver fibrosis [40]. It has been reported that fibroblasts in the lung can secrete CXCL10, which affects the polarization of alveolar macrophages in response to inflammatory stimuli. Culturing alveolar macrophages with conditioned medium of lung fibroblasts or exogenous addition of CXCL10 recombinant protein, promotes macrophage M1 polarization and inhibits M2 polarization [41]. In addition, hypothermia-induced CXCL10 or IL-6 induced M1 polarization of splenic macrophages and upregulated the expression of pro-inflammatory cytokines [42]. Consisted with these studies, we found that deletion of MyD88 in HSCs inhibited CXCL10 expression and secretion, while CXCL10 secreted by HSCs regulated macrophage polarization. Exogenous addition of CXCL10 recombinant protein increased the expression of iNOS, IL-6, and TNF-α in macrophage RAW264.7 cells. Furthermore, inhibition of CXCR3, the receptor for CXCL10, abolished these phenomena suggesting that CXCL10 plays an important role in promoting M1 polarization of macrophages. It has been shown that the signaling pathway of STAT1 is involved in macrophage M1 polarization [28]. In the present study, we exogenously added CXCL10 recombinant protein activated JAK/STAT1 pathway in macrophages and inhibition of STAT1 phosphorylation in macrophages also inhibited macrophage M1 polarization. These results suggest that CXCL10 induces macrophage M1 polarization depending on the JAK/STAT1 signaling pathway. Whether TLR/MyD88 involve in fibrosis through other mechanisms remains to be determined.

In conclusion, our results indicated that deletion of MyD88 in HSCs significantly attenuated liver fibrosis and liver inflammatory response (Fig. 8). Inhibition of MyD88 activation in HSCs may be a potential therapeutic target for liver fibrosis.

## Materials and methods

### Mice

MyD88^flox/flox^ and GFAP-cre/α-SMA-cre mice in a C57BL/6 background have been previously described [25–27]. Mice with a conditional knockout of MyD88 in GFAP-expressing HSCs (GFAP^MyD88−/−^ mice) or α-SMA-expressing fibroblast (α-SMA^MyD88−/−^ mice) were generated by crossing MyD88^flox/flox^ and GFAP-cre or α-SMA-cre mice. Control mice were cre-negative littermates. All mice were maintained in specific pathogen-free and humidity- and temperature-controlled microisolator cages with a 12-hour light/dark cycle in the Institute of Biophysics, Chinese Academy of Sciences. All experiments were carried out in males and treatments started when mice were 8-10 weeks old. All animal studies were performed under the approval of the Institutional Laboratory Animal Care and Use Committee of the Institute of Biophysics, Chinese Academy of Sciences.

### CCl4-induced liver fibrosis

CCl_4_ was mixed with a common corn oil (Sigma-Aldrich, St. Louis, MO, USA) at a 1:9 ratio and injected intraperitoneally (i.p.) into mice at 0.5 μl CCl_4_/g body weight twice per week for 4 weeks as described previously [43]. Five animals per group were used. Experiments were performed at least 3 times.

The CXCL10 neutralizing antibody (Invitrogen, Carlsbad, CA, USA) or control mIgG was injected i.p. at 10 mg/kg body weight twice a week. The injection starts 24 h before CCl_4_ administration and lasts for 4 weeks.

### TAA-induced liver fibrosis

To generate a TAA-induced model of hepatic fibrosis, mice were i.p. injected with TAA (Sigma-Aldrich, St. Louis, MO) in PBS (200 mg/kg) at twice/week for 12 weeks as described previously [24]. Five animals per group were used. Experiments were performed at least 3 times.

### Cell lines and treatments

The LX-2 cell line was purchased from Xiangya Medical College (Changsha, Huna, CN) and RAW264.7 cell line was obtained from the American Type Culture Collection (ATCC; Manassas, VA, USA). These cells were cultured in Dulbecco’s modified Eagle’s medium (DMEM)/ 1640 containing 10% fetal bovine serum (FBS) and 1% penicillin/streptomycin at 37 °C with 5% CO_2_. The LX-2 cells were exposed to ST2825 (20 μM) (MedChemExpress, Princeton, NJ, USA) for 2 h. After incubation, the cells were challenged with LPS (100 ng/mL) (Sigma-Aldrich, St. Louis, MO, USA) for 24 h for further analysis. RAW264.7 were treated with 100 ng/mL LPS and 10 ng/mL IFNγ to induce M1 polarization. RAW264.7 cells were treated with 10 ng/mL CXCL10 recombinant protein, 500 nM inhibitor of CXCL10 receptor CXCR3 AMG 487, 500 nM inhibitor of JAK for 24 h for further analysis.

### Blood biochemical assays

Mouse blood samples were centrifuged at 3000 rpm for 8 min to obtain serum. Serum ALT and AST levels were detected at the Vitonglihua experimental animal center (Beijing, CN).

### Histology and immunostaining

Preparation of liver tissue sections was performed as described previously [44]. The sliced liver paraffin sections were then stained with H&E and Sirius Red. For immunohistochemistry (IHC), cryostat sections were incubated with anti-F4/80, anti-CD11b and anti-Gr-1 antibodies (BD Pharmingen, San Diego, CA, USA) followed by incubation with horseradish peroxidase (HRP)-conjugated secondary antibodies. For fluorescence staining, cryostat sections were incubated with anti-collagen I, anti-MyD88 and anti-α-SMA antibodies (Abcam, Cambridge, Cambs, UK) followed by incubation with Alexa Fluor 488-conjugated or Alexa Fluor 594-conjugated secondary antibodies (Invitrogen, Carlsbad, CA, USA). Sections were evaluated under the bright-field and fluorescent microscope (DP71, Olympus, TKY, JPN).

Liver tissues of cirrhosis patient cases for Sirius red and MyD88 staining were from the First Affiliated Hospital of China Medical University (Shenyang, Liaoning, CN). The hospital provided ethical statements to confirm that the local ethics committees approved their consent procedures, and all participating patients signed an approved informed consent form. The ethical statement provided by the hospital and the protocol of the experiment were checked carefully and approved by the Ethics Committee of Beijing Jiaotong University.

### Isolation of primary HSCs

Isolation of primary HSCs was performed as previously described [43]. Filtered cells were centrifuged at 50 × *g* for 2 min to remove hepatocytes. The remaining nonparenchymal cell (NPC) fraction was collected. For HSC enrichment, the remaining NPC fraction was resuspended in 10% OptiPrep (Axis-Shield, Oslo, NOR) and placed between a bottom cushion of 15% OptiPrep and a top layer of PBS. After centrifugation at 1400 × g for 20 min, we obtained the HSC fraction at the interface between the top and intermediate layers. The purity of the HSC fraction was estimated based on the autofluorescence signal. Cell viability was examined by Trypan blue exclusion. Both the cell purity and viability were greater than 90%.

### Cellular immunofluorescence

LX-2 cells were inoculated in 96-well plates (1 × 10^3^ cells/well). For fluorescence staining, the cells were fixed in 4% formaldehyde for 15 min and permeabilized with 0.2% Triton-X 100 for 10 min at room temperature. The cells were incubated with 2% BSA to block nonspecific binding sites. Then, the cells were incubated with anti-MyD88 and anti-α-SMA antibodies (Abcam, Cambridge, Cambs, UK) followed by incubation with Alexa Fluor 488-conjugated or Alexa Fluor 594-conjugated secondary antibodies (Invitrogen, Carlsbad, CA, USA).

### Flow cytometry

Flow cytometry was performed as previously described [45]. Single-cell suspensions were prepared from liver NPCs and stained with the following directly labeled mouse-specific mAbs, Percp/Cy5.5-labeled anti-CD11b (clone M1/70) and PE-labeled anti-F4/80 (clone BM8). Antibodies were purchased from Biolegend and used at a 0.2 μg/ml concentration. Cells were collected on a FACS Calibur (BD Biosciences, San Diego, CA, USA) and analyzed by FlowJo software (TreeStar, Ashland, OR, USA).

### Transwell assay

Cell migrative and invasive abilities were assessed using the Transwell invasion chamber (Corning, Corning, NY, USA). The cells cultured in the serum-free medium were paved in the apical chamber coated and the LX-2 cell supernatant treated with LPS (100 ng/mL) and ST2825 (20 μM) was supplemented to the basolateral chamber as a chemotactic agent. After 24 h, the cells present above the membrane were removed with cotton swabs, while the cells that migrated through the membrane were stained with crystal violet and counted under an Olympus microscope (DP71, Olympus, TKY, JPN).

### Quantitative real-time polymerase chain reaction (qPCR)

Total RNA was isolated from frozen liver pieces or cells using TRIzol (TransGen Biotech, Beijing, CN). cDNA was synthesized using a Primescript RT Master Mix Kit (MedChemExpress, Princeton, NJ, USA). qPCR was performed in duplicates with a SYBR Premix Ex Taq^TM^ Kit (MedChemExpress, Princeton, NJ, USA). The primer sequences are listed in Supplementary Table 1. Data were analyzed using the 2^−ΔΔCt^ method and normalized to β-actin expression.

### Western blot analysis

Tissue and cell extracts were analyzed with the following primary antibodies: anti-collagen I, anti-α-SMA, anti-MyD88 (Abcam, Cambridge, Cambs, UK), anti-p-JAK1/2, anti-JAK1/2, anti-p-STAT1, anti-STAT1 antibodies (Cell Signaling, Danvers, MA, USA) and β-actin (Santa Cruz Biotechnology, CA, USA). HRP-conjugated goat anti-mouse IgG and goat anti-rabbit IgG were used as secondary antibodies. Blots were scanned using a Clinx Science Instrument.

### ELISA

LX-2 cells were pretreated with ST2825 (20 μM) for 2 h, and then incubated with LPS (10 μg/mL) and ConA (10 μg/mL) for 24 h. After activation, the cells were rinsed with PBS and cultured in fresh serum-free medium. After 24 h, the supernatant was harvested and used for subsequent ELISA determination. CXCL10 ELISA kit was purchased from BIOSs (Beijing, CN). All tests were carried out according to the manufacturer’s instructions.

### RNA Sequencing Analysis

RNA-sequencing analyses were performed in fibrotic liver tissues from control and GFAP^MyD88−/−^ mice. Total RNA was extracted with RNeasy Mini Kit (QIAGEN, Dusseldorf, GER), and RNA-sequencing analyses were performed on the BGISEQ-500 sequencer platform by BGI (Shenzhen, Guangdong, CN). Stats package and plots with ggplot2 package in R (version 3.5) were used in principle component analysis. The raw transcriptomic reads were mapped to Nipponbare reference genome using HISAT40/Bowtie241 tools after removing adaptor sequences, reads containing polyN sequences, and low-quality reads. Normalization was performed and RESM software was used. Significantly differentially expressed genes (DEGs) were identified by setting padj <0.05, and the absolute value of log2 Ratio≥1. The Kyoto Encyclopedia of Genes and Genomes (KEGG) enrichment analysis was performed by using phyper in R. All data mining, and figure presentation were conducted on the Dr Tom network platform of BGI (http://report.bgi.com).

### Public database analysis

Gene expression data (GSE14323 profiling data) were downloaded as raw signals from the Gene Expression Omnibus (http://www.ncbi.nlm.nih.gov/geo), and were analyzed using the Geo2R function from NCBI (http://www.ncbi.nlm.nih.gov/geo/geo2r). Analysis of CXCL10 and CD86 expression were performed in clinical samples from normal and cirrhosis patients in the GSE14323 database. Differences were analyzed by the log-rank test.

### Statistical analysis

All data were expressed as the mean ± SEM and were analyzed using GraphPad Prism software. Significant differences between mean values were obtained using three independent experiments. Differences between two groups were compared using two-tailed unpaired Student’s t-test analysis. One-way ANOVA with Bonferroni correction was used for multiple comparisons. *p* < 0.05 was considered statistically significant.

### Reporting summary

Further information on research design is available in the Nature Research Reporting Summary linked to this article.

## Supplementary information

Supplemental Information

Original Data File

Reporting Summary

## Data Availability

The datasets utilized in the present study are available from the corresponding author on reasonable request.
