# Supplementary material for: MyD88 in hepatic stellate cells enhances liver fibrosis via promoting macrophage M1 polarization
Source: Cell Death Dis. 2022 Apr 28;13(4):411. doi: 10.1038/s41419-022-04802-z (PMC9051099; doi:10.1038/s41419-022-04802-z)
Supplement: Supplementary file 1 — Supplemental Information [file 41419_2022_4802_MOESM1_ESM.pdf]

## Supplemental Information

# MyD88 in hepatic stellate cells enhances liver fibrosis via promoting macrophage M1 polarization

Jie Zhang[1]*, Yu Liu[1]*, Haiqiang Chen[1], Qi Yuan[1], Jinyan Wang[2], Meng Niu[3], Lingling Hou[1], Jianchun Gu[4#], Jinhua Zhang[1#]

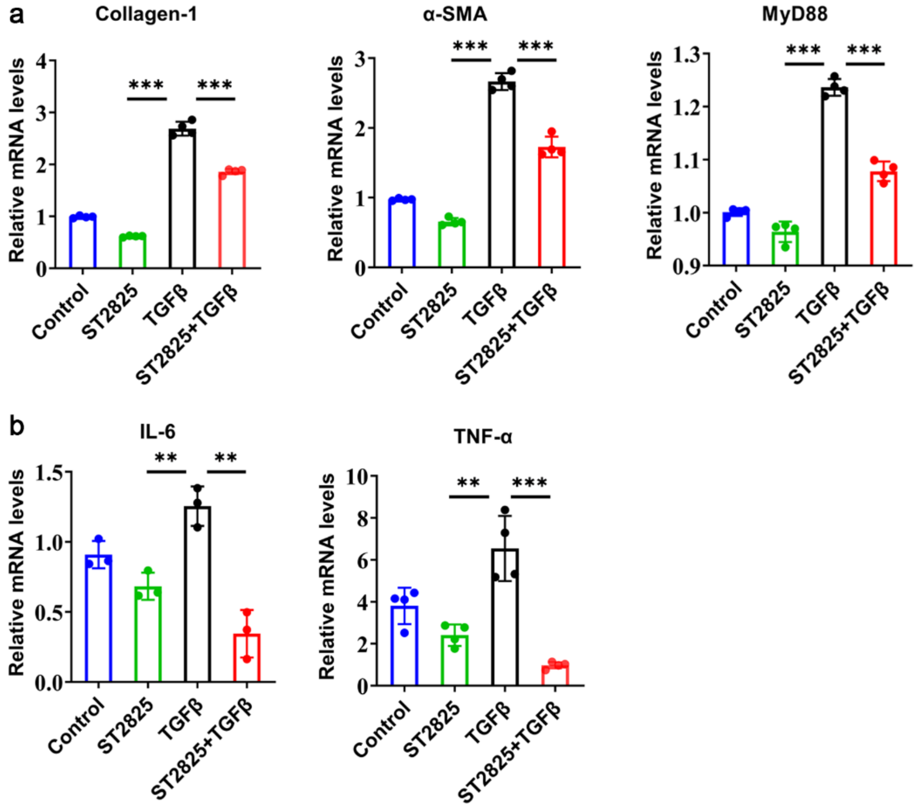


**Fig. S1. Related to Figure 4. Inhibition of MyD88 attenuates the activation and inflammatory response of hepatic stellate cells.**

LX-2 cells were activated with TGFβ (10 ng/mL) and incubated with MyD88 inhibitor ST2825 (20 μM) for 24 h. **a** The mRNA levels of Collagen-I, α-SMA and MyD88 in LX-2 cells were measured using qPCR analysis. ***$p<0.001$. **b** The mRNA levels of IL-6, and TNF-α in LX-2 cells were detected by qPCR. **$p<0.01$, ***$p<0.001$.

S2

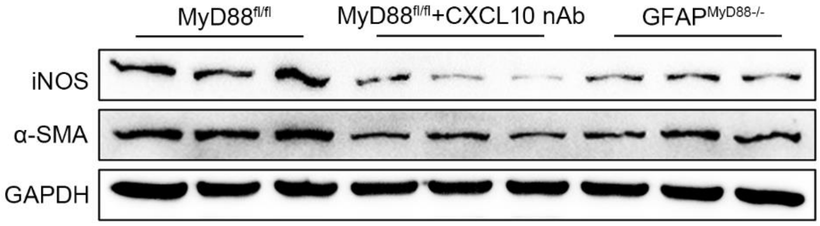


**Fig. S2. Related to Figure 7. Anti-CXCL10 treatment attenuates $CCl_4$-induced liver fibrosis.**

Groups of MyD88$^{fl/fl}$ mice (n=5 per group) were treated with $CCl_4$ and anti-CXCL10 mAb or mIgG twice a week for 2 weeks. GFAP$^{MyD88-/-}$mice were injected with $CCl_4$ at the same time. Protein levels of iNOS and α-SMA in liver tissues of mice were detected by Western blot.

Table S1. Real-time PCR primer sequences

| Gene Name | Species | Primer Sequences |
|---|---|---|
| MyD88 | human | F: AGGTAAGTAGAGCACAGATTCCTCCTA<br>R: TGTATCTGTCTGCCTGTCCATGT |
| α-SMA | human | F: CAGCACCGCCTGGATAGCC<br>R: AGGCACCCCTGAACCCCAA |
| CXCL10 | human | F: GTACGCTGTACCTGCATCAGCATTAG<br>R: CTGGATTCAGACATCTCTTCTCACCC |
| GAPDH | human | F: CCAAGGAGTAAGACCCCTGG<br>R: AGGGGAGATTCAGTGTGGTG |
| α-SMA | mouse | F: GTCCCAGACATCAGGGAGTAA<br>R: TCGGATACTTCAGCGTCAGGA |
| col1α1 | mouse | F: GCTCCTCTTAGGGGCCACT<br>R: CCACGTCTCACCATTGGGG |
| IL-6 | mouse | F: TTCTTGGGACTGATGCTGGT<br>R: CTGTGAAGTCTCCTCTCCGG |
| TNF-α | mouse | F: TGAGGTCAATCTGCCCAAGT<br>R: GGGGTCAGAGTAAAGGGGTC |
| Cre | mouse | F: GATCTCCGGTATTGAAACTCCAGC<br>R: GCTAAACATGCTTCATCGTCGG |
| iNOS | mouse | F: CGGAGATCAATGTGGCTGTG<br>R: GAAGGACTCTGAGGCTGTGT |
| IL-12p40 | mouse | F: GACATGTGGAATGGCGTCTC<br>R: TTATTCTGCCGTGCTTC |
| GAPDH | mouse | F: TGGCCTTCCGTGTTCCTAC<br>R: GAGTTGCTGTTGAAGTCGCA |

F: forward primer; R: reverse primer
