# Supplementary figures and images for: MyD88 in hepatic stellate cells enhances liver fibrosis via promoting macrophage M1 polarization
Source: Cell Death Dis. 2022 Apr 28;13(4):411. doi: 10.1038/s41419-022-04802-z (PMC9051099; doi:10.1038/s41419-022-04802-z)

Original Western Blot

Fig. 1f

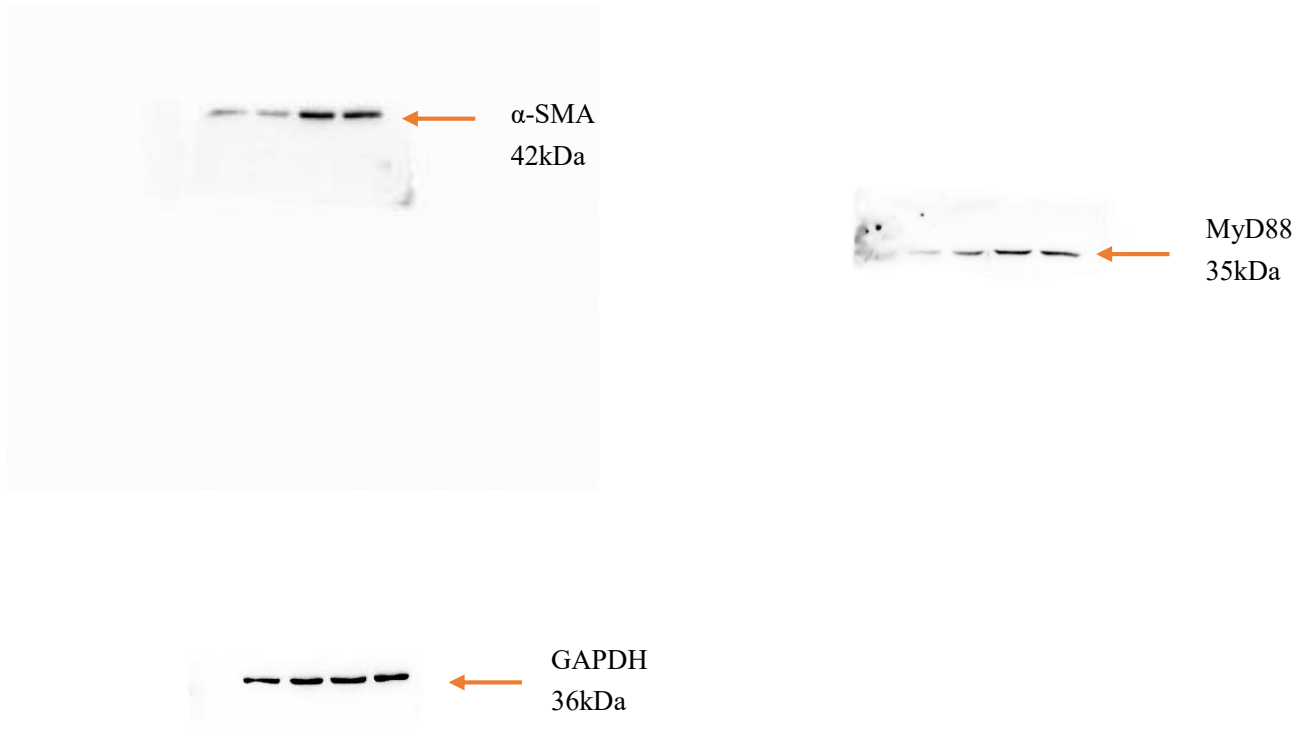

Fig. 4c

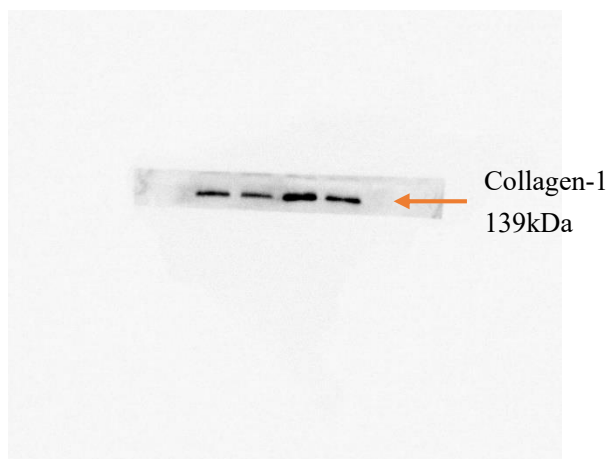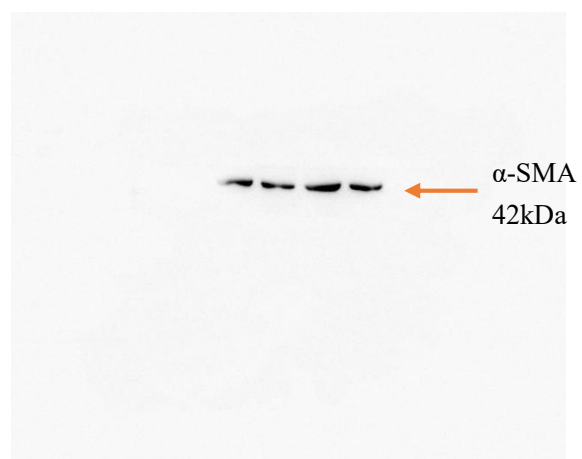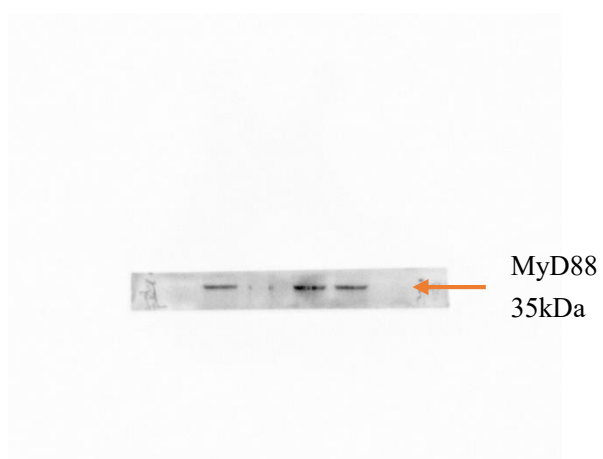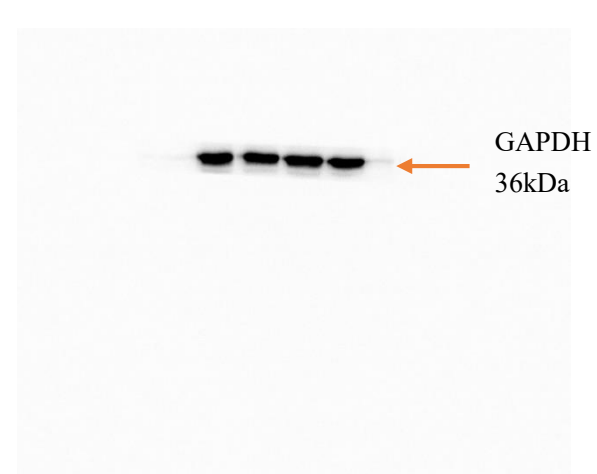

Fig. 6d

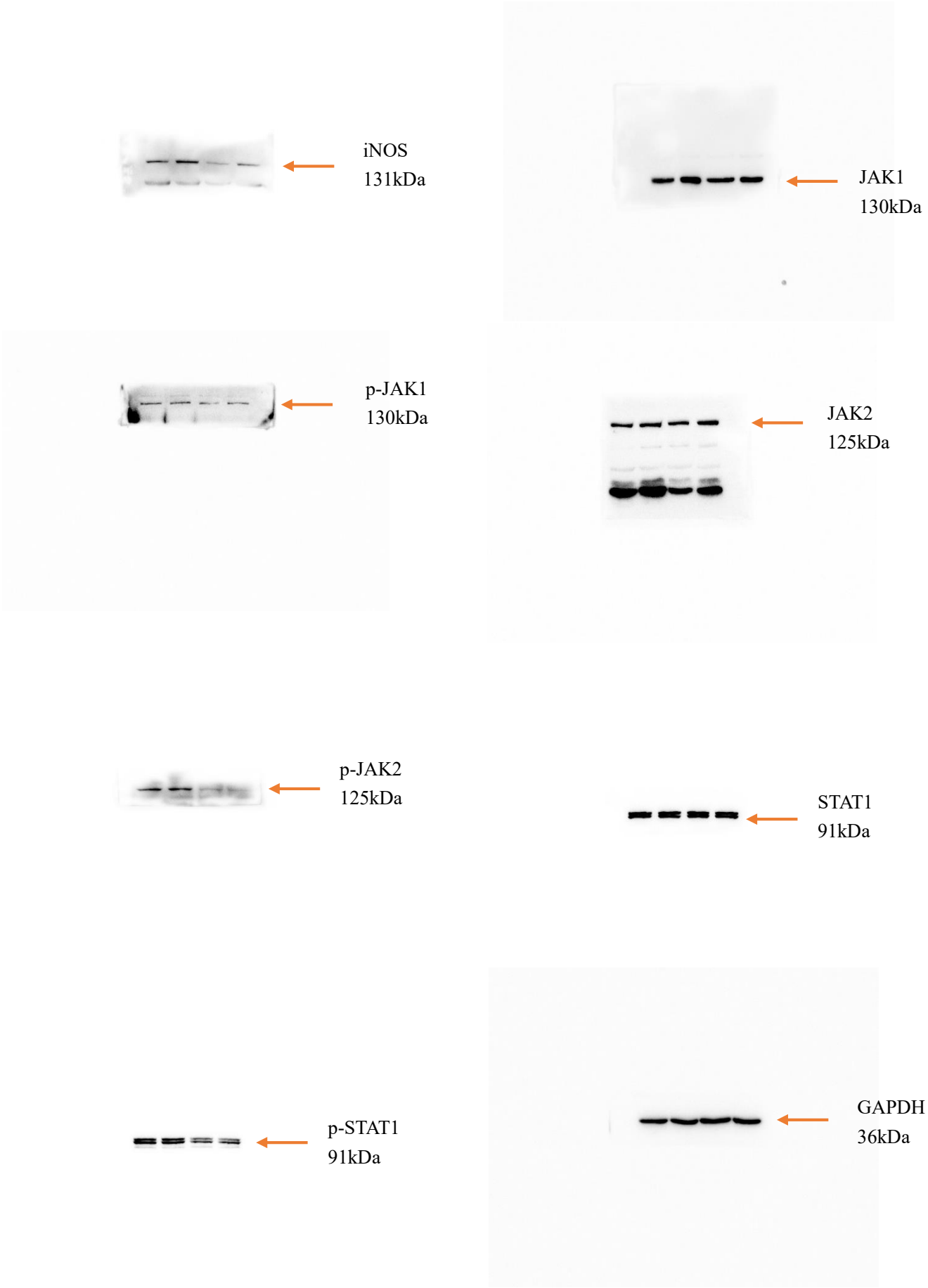

Fig. 6f

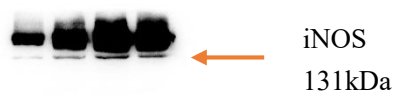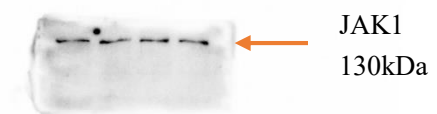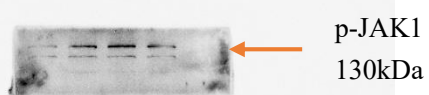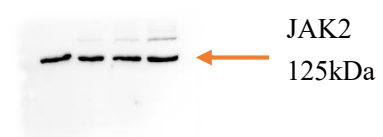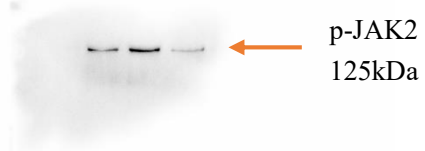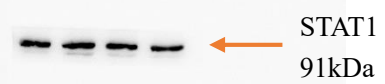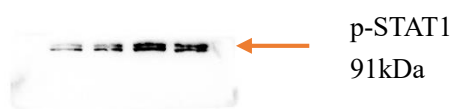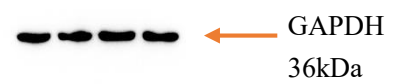

Fig. 6g

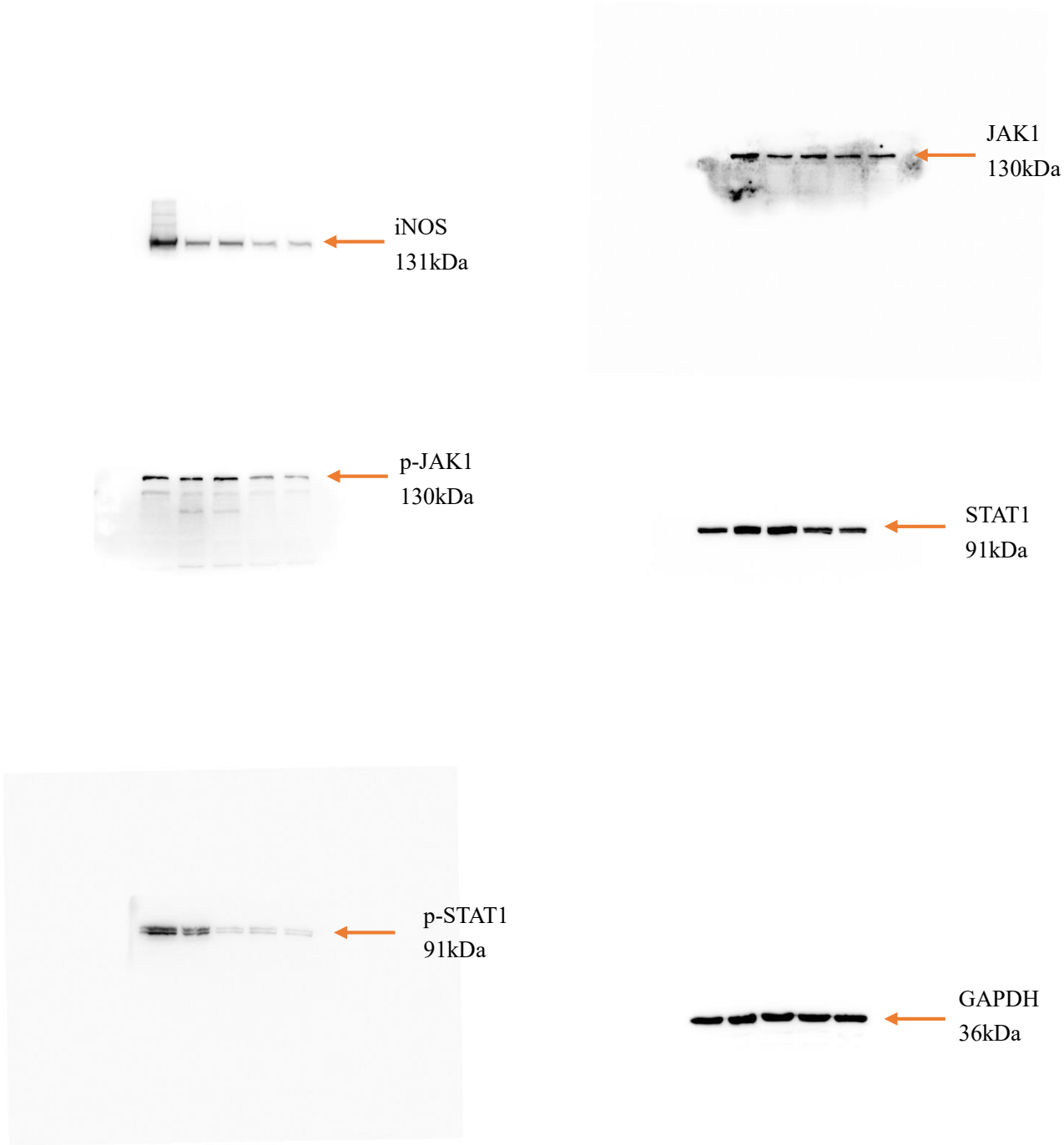

Fig.S1

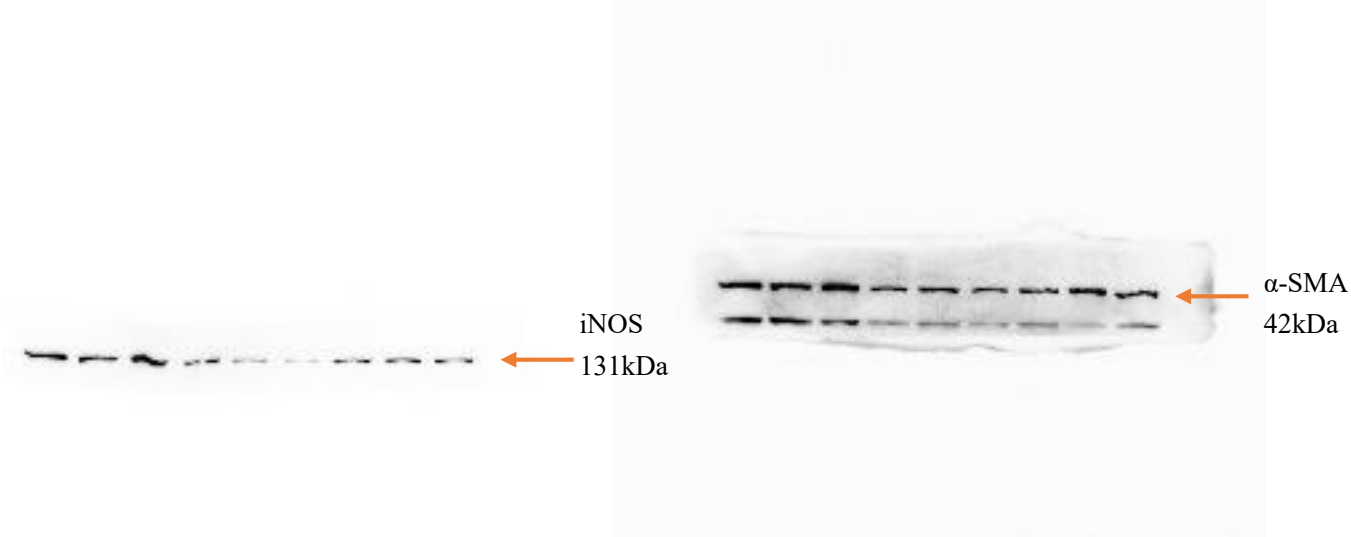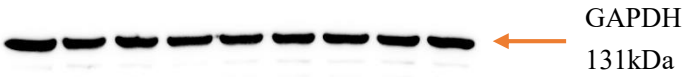

Supplement: Supplementary file 2 — Original Data File [file 41419_2022_4802_MOESM2_ESM.pdf]
